# Efficacy of selamectin, spinosad, and spinosad/milbemycin oxime against the KS1 *Ctenocephalides felis* flea strain infesting dogs

**DOI:** 10.1186/1756-3305-6-80

**Published:** 2013-03-25

**Authors:** Michael W Dryden, Patricia A Payne, Vicki Smith, Thomas C Berg, Melanie Lane

**Affiliations:** 1 Department of Diagnostic Medicine/Pathobiology, Kansas State University, Manhattan, KS 66506, USA; 2 Zoetis (formerly Pfizer Animal Health, Inc.), 333 Portage Street, KZO-300-306SW, Kalamazoo, MI, 49007, USA

**Keywords:** Flea, Ctenocephalides felis, Selamectin, Spinosad, Efficacy

## Abstract

**Background:**

A study was conducted to evaluate and compare the efficacy of selamectin, spinosad, and spinosad/milbemycin oxime against the KS1 strain of *Ctenocephalides felis* on dogs.

**Methods:**

Forty-eight dogs were selected for the study and two batches of 24 were blocked and allocated randomly to treatment groups and flea count times. There were four treatment groups of 12 dogs each: negative control, topical selamectin, oral spinosad/milbemycin oxime, and oral spinosad. Each dog was infested with 100 fleas on Days -2, 7, 14, 21 and 28. Within each treatment group, six dogs were flea counted at 24 hours and six at 48 hours after treatment or post-infestation. On Day 0, dogs received a single treatment of the appropriate drug according to the approved commercial label.

**Results:**

Efficacy of selamectin against an existing flea infestation was 60.4% and 91.4% at 24 and 48 hours, respectively, whereas spinosad/milbemycin oxime and spinosad were 100% at both time points. All products were >90% effective within 24 hours after subsequent infestations on Days 7, 14 and 21. Following the Day 28 flea infestation, selamectin was 93% and 95.7% effective at 24 and 48 hours, respectively. Whereas the efficacy of spinosad/milbemycin oxime following the day 28 infestation was 84.7% and 87.5% at 24 and 48 hours, respectively and spinosad alone was 72.9% and 76.3% effective at 24 and 48 hours, respectively.

**Conclusions:**

After initial application, the two oral spinosad products had a more rapid onset of flea kill than topical selamectin which took up to 48 hours to control (>90%) the existing infestation. However, for subsequent weekly flea infestations selamectin had similar or better efficacy than spinosad or spinosad/milbemycin oxime at 24 and 48 hours after infestation. Spinosad/milbemycin oxime and spinosad were >90% effective against the KS1 strain from Day 1 to Day 23. Whereas, selamectin was >90% effective against the KS1 strain of *C. felis* from Day 2 to Day 30.

## Background

Fleas are clinically important parasites of domestic pets being responsible for the production of allergic dermatitis, serving as vectors of various bacterial pathogens and are the intermediate hosts for filarid and cestode parasites and occasionally cause anemia and death [1,2]. When evaluating the efficacy of a flea adulticide a veterinarian needs to consider the initial speed of kill and residual speed of kill of the product [3]. Initial speed of kill refers to how rapidly a product kills the existing flea population on a pet once the product is administered. Initial speed of kill is important to provide the pet rapid relief from the existing infestation. Residual speed of kill refers to how rapidly a product can kill newly acquired fleas days or weeks after the product was administered [3,4]. When a dog or cat enters premises with emerging fleas, it is important to kill these fleas as rapidly as possible after they jump on the pet. A residual flea adulticide that can kill these newly acquired fleas quickly can likely decrease the transmission of vector borne diseases, assist in the management of flea allergy dermatitis, and reduce the likelihood that fleas can survive long enough to produce viable eggs [4]. Thus, while initial speed of flea kill is important, the residual speed of kill of a product is critical to the success of a flea control program.

The purpose of this study was to evaluate and compare the initial and residual speed of kill of a selamectin topical formulation, a spinosad/milbemycin oxime oral tablet and spinosad only oral tablet against the KS1 *Ctenocephalides felis* flea strain on dogs. The cat flea, *C. felis* is generally the most common flea species parasitizing dogs worldwide [2]. Both compounds being evaluated are systemically active with selamectin being a topically applied transdermally absorbed macrocyclic lactone with broad-spectrum activity against numerous external and internal parasites [5]. Spinosyns are a group of natural products produced by fermentation of the actinomycete, *Saccharopolyspora spinosa.* The two most abundant components produced during the fermentation process are Spinosyns A and D, which are the insecticidal active components of orally administered spinosad [6]. The flea strain that was used in this study was the KS1 cat flea strain that has been maintained as a closed colony at Kansas State University since 1990. This strain was selected because previous studies have indicated that the KS1 strain has some level of resistance or reduced susceptibility to a variety of insecticides [4,7-12].

## Methods

### Animals and housing

Fifty four purpose bred mongrel dogs (28 m:26f) were housed in individual runs. No drugs, baths, shampoos, or pesticides were administered to the dogs during the preconditioning phase or during the course of the study, other than what was described in the protocol. All animal care procedures conformed to guidelines established by the Institutional Animal Care and Use Committee at Kansas State University (IACUC # 3115). Physical examinations were performed by a licensed veterinarian on all dogs prior to enrollment into the study and all dogs were determined to be healthy. Examinations included evaluation of rectal temperature, thoracic auscultation, skin and hair coat assessment, and the assessment of the general physical condition of each dog.

### Animal selection and randomization

Due to space restrictions, dogs were enrolled in two separate batches: 28 in batch one and 26 in batch two. On Day -7, dogs (7–8 months of age and 13.9-20.3 kg) were infested with 100 adult cat fleas, *C. felis*, (KS1 strain) 1 to 5 days post emergence. On Day -6, flea comb counts were performed to assess the ability of dogs to maintain infestations. Dogs were combed with a fine-toothed flea comb having 12–13 teeth/cm. The entire dog and the entire length of the hair coat were combed continuously for a minimum of 10 minutes. If <5 live fleas were encountered in the first 10 minutes, combing was terminated. If ≥5 live fleas were encountered in the first 10 minutes, the animal was combed for an additional 10 minutes.

The 24 dogs in each batch with the highest Day -6 flea counts and that were deemed in good general health by physical examination were selected. In each batch, dogs were randomly assigned to treatments, times of flea counts and pens using a randomized complete block design with a two-way factorial structure. Treatment and time of flea counts were the two factors and blocking was done using the pre-treatment host suitability flea counts on Day -6. Within blocks, animals were randomly assigned to treatments and times of flea counts. Animals within the same block were randomly assigned to pens near each other. Personnel conducting flea combing and flea counts were blinded to treatment groups.

### Treatments

Treatment groups (12 dogs/group) were comprised of non-treated controls (TO1), selamectin (6 – 12 mg/kg) (Revolution^®^; Pfizer Animal Health) topical solution (TO2), spinosad (30 – 60 mg/kg) + milbemycin oxime (0.5-1.0 mg/kg) (Trifexis^®^; Elanco) oral tablet (TO3) and spinosad (30 – 60 mg/kg) (Comfortis^®^; Elanco) oral tablet (TO4). Products were applied according to label directions and dogs were observed following treatment for any adverse events associated with the treatments.

### Efficacy evaluations

To evaluate the ability of the formulations to eliminate an existing flea infestation, all dogs were infested with 100 adult fleas on Day -2 and treatments were applied on Day 0. Initial speed of kill was determined by removing live fleas from 6 dogs in each treatment group at 24 hours, and 6 dogs at 48 hours post-treatment. Residual activity was determined by reinfesting dogs with 100 adult fleas on Days 7, 14, 21 and 28 post-treatment and then removing live fleas from 6 dogs in each treatment group at 24 hours and 6 dogs at 48 hours post-reinfestation. Fleas were removed using the previously described flea combing procedure.

### Data analysis

The individual animal was the experimental unit and all treatment comparisons were carried out at the 5% significance level (two-sided). The 24- and 48-hour flea counts from the infestation of Day 7 onwards were log (count +1) transformed prior to analysis and analyzed using a general linear mixed model for repeated measures. The statistical model included the fixed effects of treatment, time of flea count, day of infestation and all two- and three-way interactions. The random effects included batch, block within batch, animal and residual. Log (count +1) transformed flea counts from the Day -2 infestations were analyzed separately using a general linear mixed model. The fixed effects were treatment, time of flea count and treatment by time of flea count interaction and the random effects were batch, block within batch and residual. Back-transformed geometric least squares means (GLSM), 95% confidence intervals, minimums and maximums were calculated by treatment group, time of flea count and day of infestation.

The% reduction in flea counts compared to respective control at 24 hours and 48 hours after treatment and each subsequent infestation for T02, T03, and T04 was calculated as:

% reduction = 100 * [GLSM(T01) – GLSM(T0X)]/GLSM(T01). If the treatment effect or any interaction involving treatment was significant then the treatment comparisons at each time of flea count and day of infestation was conducted.

## Results

Non-treated control dogs maintained adequate flea infestations throughout the study with geometric mean flea counts ranging from 24.40 to 51.46 (Table 1). The spinosad-based formulations provided 100.0% efficacy within 24 hours of treatment (Table 1). Selamectin provided 60.4% and 91.4% efficacy with 24 and 48 hours of treatment, respectively. The efficacy 48 hours after infestations on Days 7, 14 & 21 were equivalent for all three products (Table 1). Following the Day 28 flea infestation selamectin was 93% and 95.7% effective at 24 and 48 hours, respectively. Whereas, the efficacy of spinosad/milbemycin oxime following the Day 28 infestation was 84.7% and 87.5% at 24 and 48 hours, respectively and spinosad alone was 72.9% and 76.3% effective at 24 and 48 hours, respectively (Table 1).

**Table 1:** Geometric mean flea counts and percent efficacy relative to nontreated controls for dogs treated with a selamectin topical spot-on, a spinosad/milbemycin oxime oral tablet or a spinosad only oral tablet

	**Day 0**		**Day 7**		**Day 14**		**Day 21**		**Day 28**	
**Treatment**^1^	**Mean # of fleas**^2,3^	**% control**^4^	**Mean # of fleas**	**% control**	**Mean # of fleas**	**% control**	**Mean # of fleas**	**% control**	**Mean # of fleas**	**% control**
	**24 hours post-treatment or infestation**									
Non-treated Control	26.38^a^		46.39^a^		35.99^a^		28.85^a^		51.46^a^	
Selamectin	10.45^a^	60.4	0.12^b^	99.7	1.52^b^	95.8	0.62^b^	97.9	3.62^b^	93.0
Spinosad-Milbemycin	0.00^b^	100.0	0.00^b^	100.0	0.12^c^	99.7	1.29^b,c^	95.5	7.85^b^	84.7
Spinosad	0.00^b^	100.0	0.00^b^	100.0	0.12^c^	99.7	2.19^c^	92.4	13.96^b^	72.9
	48 hours post-treatment or infestation									
Non-treated Control	36.25^a^		38.71^a^		24.40^a^		33.81^a^		30.81^a^	
Selamectin	3.10^b^	91.4	0.00^b^	100.0	0.12^b^	99.5	0.12^b^	99.6	1.33^b^	95.7
Spinosad-Milbemycin	0.00^c^	100.0	0.00^b^	100.0	0.00^b^	100	0.41^b^	98.8	3.86^b,c^	87.5
Spinosad	0.00^c^	100.0	0.00^b^	100.0	0.26^b^	98.9	0.44^b^	98.7	7.31^c^	76.3

^1 ^Each of 6 dogs in the control group received no treatment. Each of 6 dogs in the Selamectin topical spot-on, Spinosad/Milbemycin oxime oral tablet or a Spinosad only oral tablet groups were administered the formulations according to label directions on Day 0.

^2 ^Each dog was infested with 100 adult *Ctenocephalides felis *from the KS1 strain on days -2, 7, 14, 21 & 28.

^3 ^Geometric mean # of fleas recovered from dogs per treatment group.

^4^% control = ((geometric mean count control -geometric mean count treatment)/ geometric mean count control)) × 100.

^a,b,c ^geometric means within a column with unlike letter superscripts are significantly different (P <0.05).

There were no adverse events associated with treatments in this study.

## Discussion

This study demonstrated that the oral spinosad formulations provided rapid initial speed of kill against the KS1 flea strain, with 100% efficacy within 24 hours of treatment. However, the initial speed of kill of selamectin was not as rapid; selamectin provided 60.4% and 91.4% reductions in flea populations within 24 and 48 hours of treatment, respectively.

The results for the spinosad formulations in this study were similar to several previous studies where the efficacy was also 100% within 24 hours of oral administration to dogs [12-14]. The results for the speed of kill of selamectin in this study are also comparable to previous studies where efficacy ranged from 83.7% at 24 hours to 100% at 48 hours [15-17]. It is likely that the slower initial speed of kill of selamectin as compared to spinosad is related to the need to be absorbed across the skin to reach effective blood levels.

While the initial speed of kill of selamectin was slower than the spinosad formulations, the residual speed of kill of selamectin against the KS1 flea strain was similar to or better than the spinosad-based oral formulations. The spinosad/milbemycin oxime- and spinosad-based formulations were >90% effective against the KS1 strain from Day 1 to Day 23. Whereas, selamectin was >90% effective against the KS1 strain of from Day 2 to Day 30. The residual speed of kill of selamectin in this study was similar to that seen in previous studies. In two studies the efficacy of selamectin on dogs 48 hours following infestations on Day 28 ranged from 99.2% to 100% [16,18].

The residual speed of kill of spinosad in this study contrasts with some previous reports. In a study where dogs were fed and then given spinosad at the minimal dose of 30 mg/kg, efficacy 48 hours after the Day 28 flea infestation ranged from 99.1-99.5% [6]. In a study evaluating and comparing the spinosad and spinosad/milbemycin oxime tablet formulations administered to dogs, both formulations provided 100% efficacy 48 hours after the Day 28 flea infestation [14]. However, in a different study, the efficacy of spinosad administered to dogs 24 and 48 hours after the Day 28 infestation was 85.0% and 89.0%, respectively [19]. These data are further contrasted with two previous studies evaluating the efficacy of spinosad using the KS1 flea strain where the efficacy 24 hours after the Day 28 infestation was only 22.1% and 32.5% [12]. It is interesting to note that in the current study using the same flea strain, the efficacy 24 hours following the Day 28 flea infestation was 72.9% and 84.7% for the spinosad and spinosad/milbemycin oxime formulations, respectively. It is unknown why this difference in efficacy was seen between these two studies.

Several studies have demonstrated that the KS1 flea strain has reduced susceptibility or outright resistance to carbaryl, chlorpyriphos, fenthion, fipronil, imidacloprid, permethrin, pyrethrins, and spinosad [4,7-12]. While these insecticides have performed poorly as residual insecticides against the KS1 strain, dinotefuran, metaflumizone and selamectin topical spot-on formulations have demonstrated excellent residual efficacy against this strain [3,11,12,20]. The good residual efficacy of selamectin against the KS1 flea strain was again demonstrated in this current study.

While laboratory studies are important and often necessary to evaluate and compare the efficacy of flea products, the performance of the products under natural in-home situations is equally important. A large scale clinical field trial was conducted in the U.S. where spinosad was administered orally to 330 dogs and selamectin was applied topically to 140 dogs [21]. By Days 60 and 90 of the 3 month trial, the percent reduction in flea numbers on the dogs were 99.7% and 99.9% for the spinosad-treated dogs and 97.9% and 98.9% for the selamectin-treated dogs, respectively [21]. In another three month clinical field trial conducted in France, the percent reduction in flea numbers on the dogs on Days 60 and 90 were 99.6% and 99.6% for the spinosad-treated dogs and 97.8% and 98.2% for the selamectin-treated dogs, respectively [22]. Both these clinical field trials demonstrated that selamectin and spinosad were highly effective in eliminating flea infestations on client owned dogs.

## Conclusions

Topically applied and systemically active selamectin was ≥90% effective against the KS1 strain on dogs from Day 2 to Day 30. Whereas, the oral spinosad/milbemycin oxime tablet and spinosad only tablets provided ≥ 90% flea reduction in KS1 flea populations on dogs from Day 1 to Day 23. While the two oral spinosad products had a more rapid onset of flea kill, the topical selamectin formulation had similar or slightly better residual efficacy than spinosad and spinosad/milbemycin oxime.

## Competing interests

MWD has served as a consultant and has been sponsored to lecture by Zoetis (formerly Pfizer Animal Health) and Elanco, manufacturers of Revolution^®^, Trifexis^®^ and Comfortis^®^, products that were evaluated in these investigations. TCB and ML are current employees of Zoetis.

## Authors’ contributions

MWD aided in the design of the study, served as study investigator and drafted the manuscript. VS coordinated and supervised data collection and entry and revision of manuscript; TCB and ML assisted in design of study, monitoring of study and manuscript revision. All authors reviewed and approved the final manuscript.
